# The role of physical cues in the development of stem cell-derived organoids

**DOI:** 10.1007/s00249-021-01551-3

**Published:** 2021-06-13

**Authors:** Ilaria Tortorella, Chiara Argentati, Carla Emiliani, Sabata Martino, Francesco Morena

**Affiliations:** Department of Chemistry, Biology and Biotechnology, University of Perugia, Via del Giochetto, 06123 Perugia, Italy

**Keywords:** Mechanotransduction, Mechanosensing, Pluripotent stem cells, Adult stem cells, Stem cells specification

## Abstract

Organoids are a novel three-dimensional stem cells’ culture system that allows the in vitro recapitulation of organs/tissues structure complexity. Pluripotent and adult stem cells are included in a peculiar microenvironment consisting of a supporting structure (an extracellular matrix (ECM)-like component) and a cocktail of soluble bioactive molecules that, together, mimic the stem cell niche organization. It is noteworthy that the balance of all microenvironmental components is the most critical step for obtaining the successful development of an accurate organoid instead of an organoid with heterogeneous morphology, size, and cellular composition. Within this system, mechanical forces exerted on stem cells are collected by cellular proteins and transduced via mechanosensing—mechanotransduction mechanisms in biochemical signaling that dictate the stem cell specification process toward the formation of organoids. This review discusses the role of the environment in organoids formation and focuses on the effect of physical components on the developmental system. The work starts with a biological description of organoids and continues with the relevance of physical forces in the organoid environment formation. In this context, the methods used to generate organoids and some relevant published reports are discussed as examples showing the key role of mechanosensing–mechanotransduction mechanisms in stem cell-derived organoids.

## Organoids

**Fig. 1:**
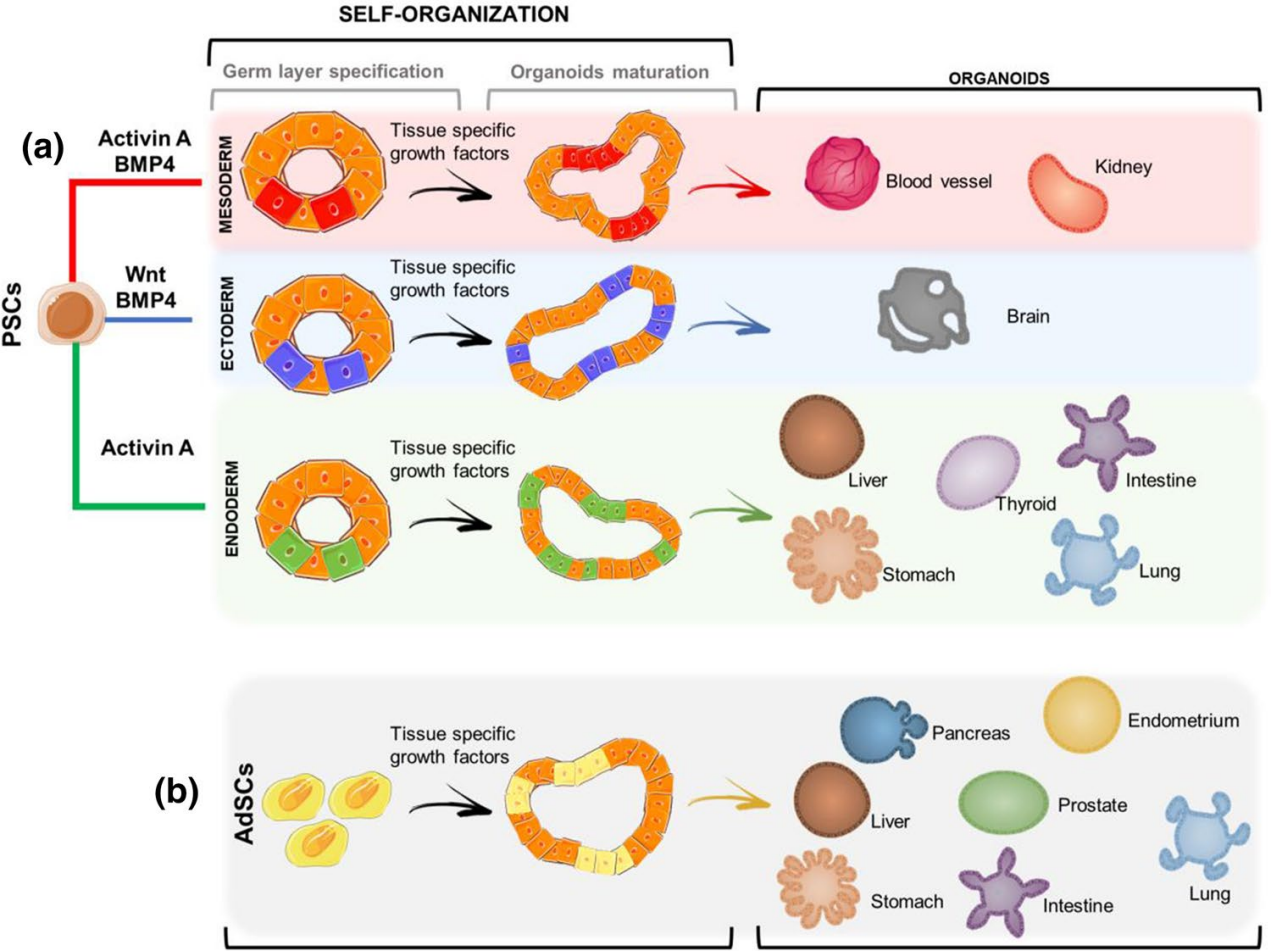
Schematic of organoids developmental process. **a** Pluripotent stem cells (PSCs) require a first step of induction toward a specific germ-layer (Activin-A and BMP4 for Mesoderm, Wnt and BMP4 for ectoderm and Activin A for Endoderm). Germ-layer specification is then followed by organoids maturation that occurs as a result of self-organization and tissue-specific growth factors leading to mature organoids: blood vessel and kidney (mesoderm), brain (ectoderm), liver, thyroid, intestine, stomach, lung (endoderm). **b** Adult stem cells (AdSCs) are tissue-specific therefore organoids specification and maturation is obtained through tissue-specific growth factors and self-organization (e.g. pancreas, endometrium, liver, prostate, stomach, intestine, lung). Bone morphogenetic protein 4 (BMP4); wingless-related integration site (Wnt)

**Fig. 2:**
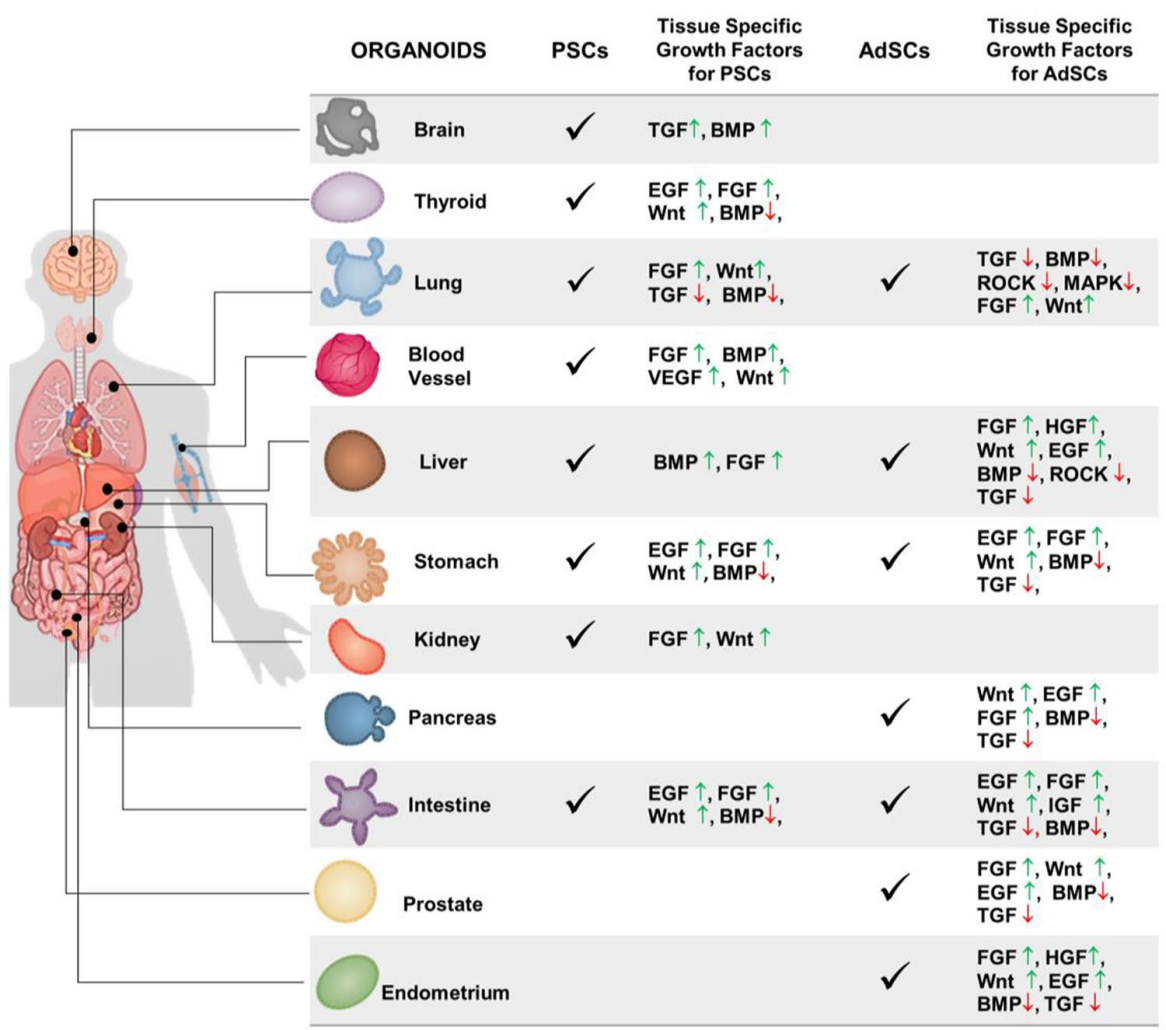
Origin and tissue-specific growth factors for the generation of human organoids. Pluripotent Stem Cells (PSCs) and adult stem cells (AdSCs) are guided toward the maturation of a specific organoid by the introduction in culture of specific growth factors that activate (arrow up ↑, green) or repress (arrow down ↓, red) particular signaling pathways (Kim et al. 2020). Bone morphogenetic protein (BMP); epidermal growth factor (EGF); fibroblast growth factors (FGF); hepatocyte growth factor (HGF); insulin-like growth factor (IGF); microtubule associated protein kinase (MAPK); RHO-associated protein kinase (ROCK); transforming growth factor (TGF); vascular endothelial growth factor (VEGF); wingless-related integration site (Wnt)

In recent decades, there has been significant advancement of three-dimensional (3D)-cell culture systems to address the limitations of two-dimensional (2D) culture systems and to better mimic tissue structure and functionality. It is now commonly recognized that cells grown in 3D environments develop more specific biological multicellular structures than cells in 2D cultures, which typically acquire a monolayer morphology (Argentati et al. 2020a). In this context, stem cells, due to the staminal properties of self-renewal and differentiation toward cell types from multiple lineages, have been considered as useful tool for the building of faithful 3D models. When cultured in an appropriate environment, stem cells accomplish their intrinsic developmental programs, which result in self-organization and generation of biologically relevant 3D structures that recapitulate in vitro several features of tissues and organs and are therefore called “organoids” (Brassard and Lutolf 2019) (Figs. 1, 2).

Organoids technology takes advantage of the different characteristics of pluripotent stem cells (PSCs, both Embryonic Stem Cells and induced Pluripotent Stem Cells) and multipotent stem cells (Adult Stem Cells, AdSCs) to create 3D structures that could serve as in vitro models of different organs; therefore, offering the opportunity to observe important biological phenomena such as embryonic development and tissue regeneration and to develop personalized disease models through the building of patient-derived organoids (Lancaster and Huch 2019; Takahashi 2019; Schutgens and Clevers 2020; Zheng and Fu 2021).

On one hand, PSCs can differentiate toward all three germ layers (Endoderm, Ectoderm, Mesoderm) and are used for building more complex organoids useful for studying the embryonic development and are needed when the organ that has to be modeled is not easily accessible (e.g., the brain) (Brassard and Lutolf 2019; Liu et al. 2021; Yu et al. 2021). On the other hand, AdSCs, due to the more limited differentiation capability, are mostly used to generate organoids of their tissue of origin. AdSCs also offer the advantage of being isolated directly from patient’s biopsies thus making them a valuable tool for disease modeling and personalized medicine purposes. While the building of PSCs-derived organoids requires the reprogramming of somatic differentiated cells isolated from patients followed by expansion and differentiation, the use of AdSCs permits the production of healthy and diseased tissues in a shorter time: as a result, the latter allows a more manageable expansion of models from patients, potentially facilitating personalized medicine (Rossi et al. 2018; Lancaster and Huch 2019; Schutgens and Clevers 2020).

The generation of organoids requires also the addition of specific growth factors into the stem cell culture medium in the appropriate amount and spatiotemporal way. For instance, the step of germ-layer specification for PSCs is obtained through Activin A (Endoderm), Activin A and Bone Morphogenetic Protein 4 (BMP4, Mesoderm) and WNT + PBM4 (Ectoderm), which is then followed by a step in which tissue-specific growth factor cocktails and molecules activate particular signaling pathways, such as WNT and Fibroblast Growth Factors (FGF) (Yin et al. 2016; Lancaster and Huch 2019; Kim et al. 2020)(Figs. 1a, 2). The latter step allows the induction and maturation of organoids and is common also to the AdSCs-derived organoids maturation process (Figs. 1b, 2).

All steps of differentiation protocols aim at supplying stem cells with a range of biochemical and biophysical signals that mimic the in vivo stem cell niche, which is essential to create a good organoid model (Figs. 1, 2). This correlates with the concept that tissue and organ development, including cell specification, differentiation, survival, and proliferation, is heavily reliant on complex networks and coordination of cell-to-cell, and cell-Extracellular Matrix (ECM) interactions, as cooperative cell activity differs significantly from individual cell behavior (Dahl-Jensen and Grapin-Botton 2017).

The strict dependence of organoids formation and biochemical and biophysical environmental conditions is a crucial aspect that contributes deeply to the successful development of accurate models but also inevitably introduces a certain grade of randomness into organoids formation, resulting in heterogeneous morphology, size, and cellular composition (Hofer and Lutolf 2021). The concept of reproducibility in organoids research is one of the major obstacles for their scalability and full use in pre-clinical applications, hence fine-tuning the culture microenvironment is unquestionably essential for the advancement of this technology (Rossi et al. 2018; Lehmann et al. 2019; Brassard and Lutolf 2019; Hofer and Lutolf 2021). Indeed, there are hurdles that still need to be fully managed as low-maturation level, small size (not more than a few millimeters), morphological variability and lack of fundamental biological components like vascularization and immune system (Lancaster and Knoblich 2014; Shen 2018; Holloway et al. 2019; Brassard and Lutolf 2019; Zahmatkesh et al. 2021).

**Fig. 3:**
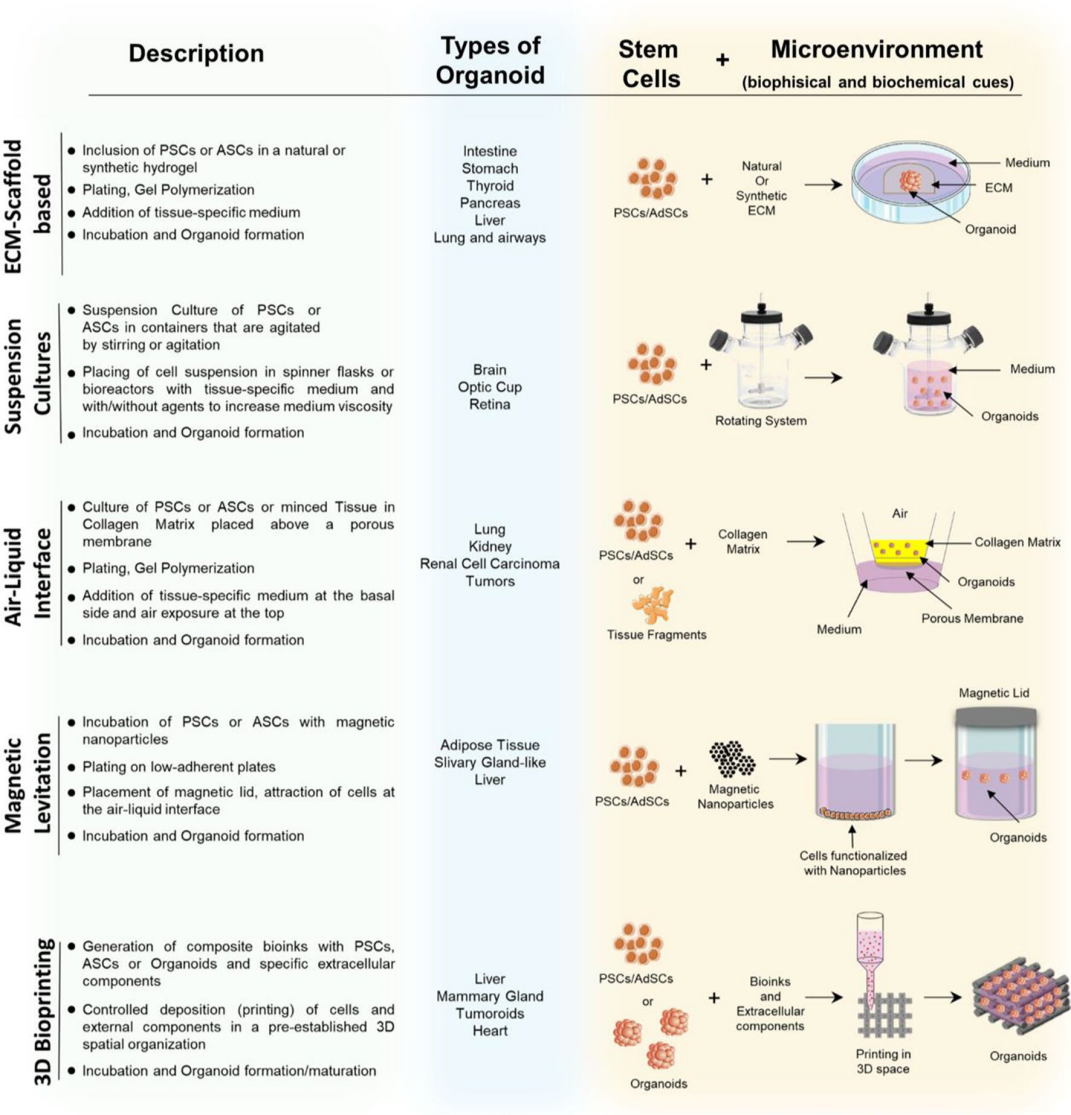
Conventional methods for organoids generation. Schematization of the main steps required in the techniques most frequently used for organoids generation: ECM-scaffold-based, suspension culture, air–liquid interface, magnetic levitation and 3D bioprinting (grey column) with related examples of produced organoids (Blue column, references in the text). Schematic representation of method used for organoids generation: biological elements (cells) and microenvironment required for organoids maturation (biophysics and biochemical components). Pluripotent stem cells (PSCs); adult stem cells (AdSCs)

The delicate balance required to maintain homogeneous organoids cultures highlights the role of the environment in controlling the cellular polarization in a context-dependent manner (Brassard and Lutolf 2019). Thus, is now widely recognized that organoids formation is deeply influenced by small changes in the culture condition (Hofer and Lutolf 2021). Therefore, all methods used for organoids generation consist in the inclusion of stem cells in an environment characterized by specific biophysical and biochemical components (Fig. 3). These elements mimic the role of the structure as a well as of soluble biomolecules in the in vivo stem cell niche, allowing for better regulation of cellular growth and differentiation and, as a result, more physiological applicable model systems that can be translated into clinical practice (Hofer and Lutolf 2021).

The commonest method currently used for the generation of organoids is the ECM-scaffold based (Shah and Singh 2017; Velasco et al. 2020). In this technique, organoids are generated by including stem cells in an environment consisting of a biophysical component, generally natural (Matrigel, Collagen, Alginate, Fibrin, Laminin) or synthetic (e.g., Polyethylene Glycol, PEG) hydrogels, and biochemical component, such as different types of soluble bioactive chemical/biological molecules (Sato et al. 2009; Kurmann et al. 2015; Workman et al. 2017; McCracken et al. 2017; Hohwieler et al. 2017; Shah and Singh 2017; Chen et al. 2017; Camp et al. 2017; Yan et al. 2018). Alternatively, organoids can be generated with the (i) *suspension culture* procedure accompanied by the use of *spinner flasks* or *rotating bioreactors*, which can be described as rotating cell culture systems (Nakano et al. 2012; Qian et al. 2018; Hoarau-Véchot et al. 2018; Przepiorski et al. 2018; Capowski et al. 2019; Velasco et al. 2020; Sander et al. 2020); (ii) Air–liquid interface (ALI*)*, where stem cells are exposed to culture medium on one side and to air on the other for maximizing the oxygen and nutrient supply (Takasato et al. 2015; Neal et al. 2018; Choi et al. 2020; Lo et al. 2020; Esser et al. 2020; Gunti et al. 2021); (iii) Magnetic levitation, which poses its bases in tagging cells with magnetic nanoparticles and then exposing them to a magnetic field that levitates them to the liquid–air interface where they aggregate and generate ECM components (Desai et al. 2017; Tseng et al. 2018; Ferreira et al. 2019; Velasco et al. 2020); (iv) 3D bioprinting, which could allow controlling the spatial positioning of cells and other biological components such as growth factors and ECM structural components (Fig. 3)(Duelen et al. 2019; Reid et al. 2019; Sun et al. 2020; Kupfer et al. 2020; Rawal et al. 2021; Yang et al. 2021).

## Organoids and mechanobiology

Mechanical forces and spatiotemporally coordinated cellular signaling patterning are now recognized as essential factors in tissues organization and acquisition of their functional adult state in vivo (Jansen et al. 2015; Weaver 2017; Mohammed et al. 2019; Argentati et al. 2019; Kim et al. 2021). The mechanical forces that regulate and act on the 3D adult tissue organization, are transmitted within the tissue by individual cells that are confined in the ECM (Humphrey et al. 2014; Stanton et al. 2019; Argentati et al. 2019; Kim et al. 2021). In this section, we will discuss the relevance of mechanobiology in organoids development. To be clear, the section starts with some notes on mechanobiology.

### Pills of mechanobiology

Over the last two decades, evidence has accumulated demonstrating how the physico-chemical properties of the cellular microenvironment, as well as the physical forces exerted by cells and tissues, are critical in the regulation of physiological conditions (such as tissue development, repair, and homeostasis, cell motility, proliferation, metabolism and differentiation) (Mammoto and Ingber 2010; Morena et al. 2017, 2020; Argentati et al. 2018, 2019; Wolfenson et al. 2019) but also pathological states (Jansen et al. 2015; Jensen et al. 2015; Alcaraz et al. 2018; Kim et al. 2019; Lee et al. 2019; Argentati et al. 2019, 2020b; Hall et al. 2020). In both contexts, cells must adapt their behavior using their capability to sense the external physical forces—*mechanosensing*—and to transduce these forces into biochemical signals—*mechanotransduction* (Trubelja and Bao 2018; Martino et al. 2018; Argentati et al. 2019). Both mechanisms collect the activity of several intracellular and extracellular components (Table 1) that, working together in a spatial–temporal manner, transmit the signaling to the cell DNA and change the cell gene expression (Trubelja and Bao 2018; Martino et al. 2018; Argentati et al. 2019; Janota et al. 2020). The most known pathways include (i) integrins—ECM—Focal adhesion (FAs) complexes—cytoskeleton—nucleoskeleton proteins (Weinberg et al. 2017; Jansen et al. 2017; Morena et al. 2017; Martino et al. 2018; Luzi et al. 2020; Argentati et al. 2021); (ii) Adherens Junctions (AJs) complexes for cell–cell interaction—cytoskeleton—nucleoskeleton proteins (Morena et al. 2017; Martino et al. 2018; Yap et al. 2018; Liebman et al. 2020). The overall interconnection also influences the behavior of neighboring cells and can remodel constantly the ECM environment through synthesis, degradation, and chemical modification processes (Humphrey et al. 2014; Stanton et al. 2019; Argentati et al. 2019).

**Table 1:** Cellular components involved in mechano-sensing and mechano-transduction processes and forces to which they respond

Location	Proteins	Mechanical forces to which proteins respond		References
ECM (Extracellular Matrix)	Collagens	Compression Elasticity Hydrostatic pressure	Tension Viscoelasticity	(Saini and Kumar 2015; Chooi and Chan 2016; Argentati et al. 2019)
	Elastin	Compression Elasticity	Tension Viscoelasticity	(Andrikakou et al. 2016; Cocciolone et al. 2018; Argentati et al. 2019)
	Fibrillin	Elasticity	Tension	(Schrenk et al. 2018; Argentati et al. 2019)
	Fibulin	Stiffness	Tension	(Nakasaki et al. 2015; Argentati et al. 2019)
	Fibronectin	Elasticity Stiffness	Tension	(Wang et al. 2016b; Martino et al. 2018; Argentati et al. 2019)
	Laminin	Shear stress		(Di Russo et al. 2017)
	Tenascin	Elasticity	Tension	(Imanaka-Yoshida and Aoki 2014; Argentati et al. 2019)
Cell Membrane	Integrins	Elasticity Hydrostatic pressure Shear Stress	Stiffness Tension	(Jang and Beningo 2019; Kechagia et al. 2019; Argentati et al. 2019)
FAs (Focal adhesion complex)	Tensin	Tension		(Argentati et al. 2019)
	Vinculin	Stiffness	Tension	(Atherton et al. 2016; Omachi et al. 2017; LaCroix et al. 2018)
	Paxillin	Stiffness	Tension	(Zhou et al. 2017; Argentati et al. 2019)
	Talin	Stiffness	Tension	(Kumar et al. 2016)
	FAK	Elasticity Stiffness	Tension	(Bell and Terentjev 2017; Argentati et al. 2019)
AJs (Adherens Juctions)	βCatenin	Compression	Shear stress	(Sheng et al. 2018; Argentati et al. 2019)
	αCatenin	Tension		(Sarpal et al. 2019)
	Cadherins	Tension		(Pannekoek et al. 2019; Argentati et al. 2019)
	ZO-1	Shear stress Stiffness	Tension	(Demaio et al. 2001; Haas et al. 2020)
	ICAM1	Viscoelasticity		(Wiesolek et al. 2020)
Cytoskeleton	F-actin	Compression Elasticity Hydrostatic pressure Shear stress	Stiffness Tension Viscoelasticity	(Galkin et al. 2012; Fan et al. 2019; Argentati et al. 2019; Wei et al. 2020)
	Microtubule	Tension Stiffness	Elasticity	(Brouhard and Rice 2018; Argentati et al. 2019; Hamant et al. 2019)
	Vimentin	Compression Stiffness	Viscoelasticity	(Charrier and Janmey 2016; Argentati et al. 2019)
	Titin	Elasticity		(Herrero-Galán et al. 2019; Argentati et al. 2019)
	Myosin II	Compression Elasticity	Tension	(Argentati et al. 2019; Fujita et al. 2019; Lou et al. 2021)
	Filamin	Stiffness		(Mezawa et al. 2016; Zhou et al. 2017; Martino et al. 2018; Argentati et al. 2019; Janmey et al. 2020)
	α-Actinin	Stiffness		(Meacci et al. 2016; Argentati et al. 2019)
	Arp2/3	Tension		(Argentati et al. 2019)
	Formin	Tension		(Zimmermann and Kovar 2019)
	Cofilin	Compression	Tension	(Gupta et al. 2016; Ikawa and Sugimura 2018)
Nucleoskeleton	Lamin A/C	Stiffness	Tension	(Chen et al. 2018; Argentati et al. 2019; Koushki et al. 2020)
	Emerin	Stiffness		(Willer and Carroll 2017; Fernandez et al. 2021)

In addition, several studies have identified molecular components involved in the mechano-sensing and—transduction processes, which respond to various mechanical forces such as *compression* (cells contract as a result of compressive forces applied from the outside to the center of cells)(Takemoto et al. 2015; Vining and Mooney 2017; Argentati et al. 2019), *tension* (external stimuli that stretch cells in opposite directions, resulting in cell elongation)(Spadaro et al. 2017; Martino et al. 2018; Rossy et al. 2018; Argentati et al. 2019), *hydrostatic pressure* (force exercised by the surrounding fluid to cells membranes, with non-directional nature influencing microtubule stability of cell cytoskeleton) (Becquart et al. 2016; Hadi et al. 2018; Pattappa et al. 2019), and *fluid shear stress* (two opposing forces applied tangentially to a cell's surface, causing changes in cell morphology and adhesion properties) (Becquart et al. 2016; Alfieri et al. 2019; Argentati et al. 2019) that in turn lead to the deformation and regulation of particular cellular environment properties including *elasticity* (the ability of an object to revert to its original shape and size after a force has been removed)(Grady et al. 2016; Argentati et al. 2019), *stiffness* (the ability of an object to resist deformation after being subjected to a force) (Islam et al. 2017; Argentati et al. 2019; Janmey et al. 2020) and *viscoelasticity* (an object’s elastic and viscous properties that contrast deformation)(Wang et al. 2016a; Argentati et al. 2019; Chaudhuri et al. 2020). (Table 1). These processes are likely activated when stem cells generate organoids (Bayir et al. 2019; Hofer and Lutolf 2021).

### Mechanical forces involved in stem cell-derived organoids formation

**Fig. 4:**
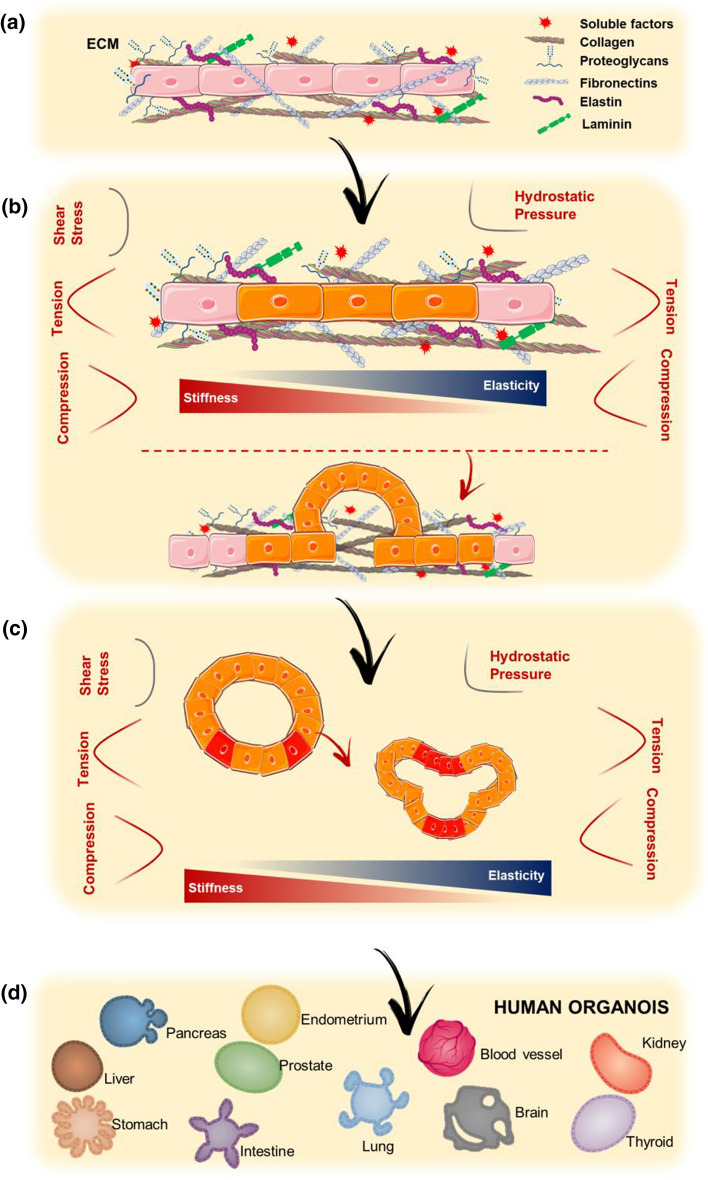
Mechanical forces and organoids formation. Schematic representation of the involvement of different mechanical and physical forces (shear stress, tension, compression, hydrostatic pressure) and environmental properties (stiffness and elasticity) in the main steps of organoids formation: **a** Stem cells are included in an environment characterized by specific chemical and structural components; **b** Different mechanical forces and environmental properties influence stem cells specification and **c** 3D self-organization; **d** All these forces and properties guide the maturation of organoids and **e** lead to the formation of specific organoids type

The engineering of the organoid microenvironment focuses on controlling diverse mechanical properties such as topography, porosity, permeability, stiffness, shape, and elasticity (Bayir et al. 2019). The combination of all these properties creates a specific microenvironment characterized by a particular set of forces that are exerted on cells indirectly via the ECM, allowing them to mechanosense and respond to these forces when forming an organoid (Fig. 4) (Dahl-Jensen and Grapin-Botton 2017; Park et al. 2019).

The identification of the appropriate pattern of forces that have to be present in culture is fundamental for steering stem cells toward the right differentiation state (Vining and Mooney 2017). Performing experiments could fully elucidate how mechanics affect particular cells or tissues in vivo, in fact several studies clarified how substrates with different mechanical properties allowed lineage-specific differentiation of stem cells. For example, matrix elasticity regulates the differentiation of Mesenchymal Stem Cells (MSCs) with the general concept that rigidity is associated with chondrogenic/osteogenic lineages and softer matrices induce neuronal or fat differentiation (Engler et al. 2006; Huebsch et al. 2010; Khetan et al. 2013; Vining and Mooney 2017; Romani et al. 2021).

Indeed, stiffness is a decisive parameter for mimicking the stem cells’ niche and it can be tuned using synthetic matrices which, in this way, offer the possibility of investigating its effect on organoids formation (Gjorevski et al. 2016). About this, new mechanical refined materials such as complex hydrogels with tunable architecture and composition that offer the possibility of precisely control the orientation of functional groups showed that the regulation of matrix viscoelasticity and gel degradability is of particular importance for a successful organoid formation and culture (Cruz-Acuña et al. 2017; Chaudhuri et al. 2020).

As far as understanding the sensing of mechanical stimuli by organoids is concerned, the clarification of how forces exactly influence organoids formation is even more difficult because they are a more complex model (compared to 2D cultures)(Chan et al. 2017) in which cells establish interactions among them and the external ECM; however, several studies explored this issue (Park et al. 2019; Bayir et al. 2019).

In this regard, in a recent study, the laboratory of H. Clevers investigated the role of matrix stiffness on the behavior of Intestinal Stem Cells (ISCs). In this work, they evidenced how Intestinal Stem Cells cultured on a stiff matrix underwent expansion enhancement, but when grown on a soft matrix differentiated and formed organoids (Gjorevski et al. 2016). In particular, first, they cultured ISCs in PEG hydrogels functionalized with the RGD (Arg-Gly-Asp) peptide and observed that ISCs expanded on the matrix with intermediate stiffness and did not on softer ones (1.3 vs 300 Pa), and afterward they used hybrid PEG hydrogels constituted by a mechanically static and a mechanically dynamic PEG to control over time the gel’s stiffness: when functionalized with RGD and laminin-111, organoids were generated only when gel stiffness was about 190 Pa and Yes-associated protein (YAP) activation was greater in these softening matrices. This study, therefore, shed light on the mechanistic role of the 3D microenvironment (Gjorevski et al. 2016).

Acknowledged the importance of mechanical forces in embryogenesis and organogenesis, the control of the biophysical microenvironment answer to the need of enhancing the reliability of organoid models. For this reason, it is now becoming clear that it is necessary to build culture systems in which is possible to produce biomechanical cues that are as physiological as possible. Recent advancement in this field is the synergic combination of organoids and organ-on-a-chip (OOC) technology: while organoids have the advantage of following self-organization, OOC offers the possibility of precisely regulate the cellular microenvironment to replicate the physiological environmental conditions (Park et al. 2019; Zheng et al. 2021). There are several OOC available on the market that employ dynamic biomechanical stimulation and that can be used to develop complex 3D tissues like spheroids, organoids, and tissues interfaces (Thompson et al. 2020).

For example, Lee et al. implemented peristaltic fluid flow in human stomach organoids; therefore, introducing contraction and stretching to mimic gastric contractions, which enabled the construction of a more solid and physiologically relevant model amenable for disease modeling and drug screening (Lee et al. 2018). To do so, human gastric organoids (GOs) generated from hPSCs were cultured in a 3D-printed device equipped with micropipettes connected to a peristaltic pump filled with FITC-dextran: following the fluorescent fluid flow, they observed a regular distribution of luminal fluid overtime and demonstrated the feasibility of GOs long-term culturing associated to nutrient and therapeutic agents delivery (Lee et al. 2018).

Berger et al. enhanced the vitality and differentiation of Midbrain organoids using a fluidic system that generated continuous laminar fluid flow (Berger et al. 2018). They compared a new milli-fluidic culture technique with the orbital shaker (commonly used for brain organoids generation) and observed that it allowed a better differentiation of Neuroepithelial Stem Cells to midbrain Dopaminergic neurons and a reduction of the inner area of cell death: interestingly, this work highlighted that different fluid dynamics have distinct effects on organoids development suggesting that the resulting diverse mechanical stimuli are involved in their homeostasis (Berger et al. 2018).

Another promising result was obtained by Tao et al. that generated iPSCs-derived Pancreas organoids in a microfluidic system that improved their viability and organ-specific functionality, like insulin secretion stimulated by glucose and higher Ca^2+^ flux (Tao et al. 2019). In accordance with the study previously proposed, this work showed that the culture of organoids under perfused conditions highlights the role of biomimetic mechanical signals in improving the functionality and maturation of islet organoids (Tao et al. 2019).

In another study, Homan et al. exploited shear stress generated with a milli-fluidic system and co-culture with endothelial cells to greatly improve the maturation of Kidney organoids managing to enhance vasculature and their tubular and glomerular compartments (Homan et al. 2019). In particular, they determined the effect of fluidic shear stress by culturing hPSCs in a chip with controlled fluid flow and observed that the vascular network formation was greatly improved under high fluidic shear stress condition compared to low, in the order of fivefold increase, indicating that shear stress is a significant cue for the vascularization of kidney organoids in vitro as it is associated to the endogenous upregulation of the vascular endothelial growth factor (Homan et al. 2019).

## Conclusion

**Fig. 5:**
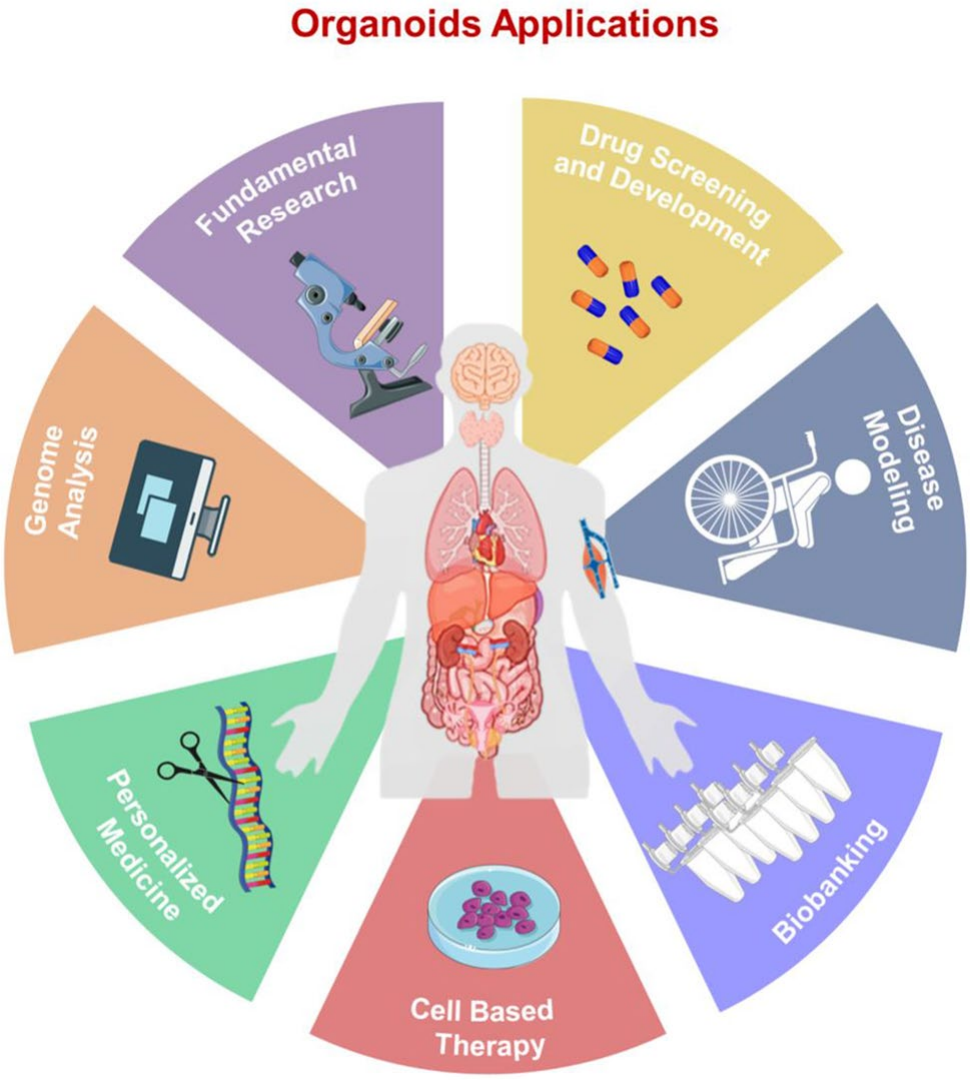
Organoids applications Organoids can be used for different biomedical applications such as Fundamental Research, Drug Screening and Development, Disease Modelling, Biobanking, Cell Based Therapy, Personalized Medicine and Genome Analysis

In this mini review, we have discussed recent key findings on the development of organoid technology (Fig. 3). In particular, we have highlighted the relevance of the environment as an active counterpart on inducing stem cells toward the generation of a specific organoid, describing the role of exogenous soluble bioactive molecules and foremost the role of the environmental physical components, and the way in which both mimic the structure and function of the stem cell niche. The role of mechanical forces has been demonstrated to significantly orchestrate the interaction of the cells with the ECM or with neighboring cells and how these interconnections are fundamental for cell functions. These roles have been confirmed also in organoids formation. Of note, to date, there are different medical applications of organoid mechanobiology-based technology such as novel drug screening, regenerative medicine application, molecular research (Fig. 5).

In this regard, many studies focused on organoids mechanobiology are ongoing and will help to elucidate the mechanism behind the biophysical aspects of organoid cultures. For instance, the European Project “Mechanoids” (Grant agreement ID: 797,621, H2020-EU.1.3.2.) aims at manipulating the mechanobiology of healthy Gut and Colorectal Cancer organoids to assess their role in disease and development processes (HORIZON 2020a) The characterization of organoids mechanobiology will be useful also for disease modeling, as planned in the project “ROMB” (Grant agreement ID: 850,691, H2020-EU.1.1.) where Retina organoids mechanobiology will be investigated to model Alzheimer’s Disease and will shed light on mechanically related neuronal diseases (HORIZON 2020b). In conclusion, despite the challenges that must be addressed, considering the advantages of ongoing technology development, organoid technology holds great promise in research and in the developing clinical translational strategies.
